# Microstructural and Mechanical Evaluation of a Cr-Mo-V Cold-Work Tool Steel Produced via Electron Beam Melting (EBM)

**DOI:** 10.3390/ma14112963

**Published:** 2021-05-31

**Authors:** Carlos Alberto Botero, Aydın Şelte, Markus Ramsperger, Giulio Maistro, Andrey Koptyug, Mikael Bäckström, William Sjöström, Lars-Erik Rännar

**Affiliations:** 1 Department of Quality Technology and Mechanical Engineering, Sports Tech Research Centre, Mid Sweden University, Kungskapensväg 8, SE-83125 Östersund, Sweden; andrey.koptyug@miun.se (A.K.); Mikael.Backstrom@miun.se (M.B.); william.sjostrom@miun.se (W.S.); Lars-Erik.Rannar@miun.se (L.-E.R.); 2 Uddeholms AB, Uvedsvägen, SE-683 85 Hagfors, Sweden; aydin.selte@uddeholm.com (A.Ş.); giulio.maistro@uddeholm.com (G.M.); 3 GE Additive, Arcam EBM Center of Excellence, Designvägen 2, SE-435 33 Mölnlycke, Sweden; markus.ramsperger@ge.com

**Keywords:** cold work, tool steel, additive manufacturing, electron beam melting, hardness, carbides, mechanical properties

## Abstract

In this work, a highly alloyed cold work tool steel, Uddeholm Vanadis 4 Extra, was manufactured via the electron beam melting (EBM) technique. The corresponding material microstructure and carbide precipitation behavior as well as the microstructural changes after heat treatment were characterized, and key mechanical properties were investigated. In the as-built condition, the microstructure consists of a discontinuous network of very fine primary Mo- and V-rich carbides dispersed in an auto-tempered martensite matrix together with ≈15% of retained austenite. Adjusted heat treatment procedures allowed optimizing the microstructure by the elimination of Mo-rich carbides and the precipitation of fine and different sized V-rich carbides, along with a decrease in the retained austenite content below 2%. Hardness response, compressive strength, and abrasive wear properties of the EBM-manufactured material are similar or superior to its as-HIP forged counterparts manufactured using traditional powder metallurgy route. In the material as built by EBM, an impact toughness of 16–17 J was achieved. Hot isostatic pressing (HIP) was applied in order to further increase ductility and to investigate its impact upon the microstructure and properties of the material. After HIPing with optimized protocols, the ductility increased over 20 J.

## 1. Introduction

Metal additive manufacturing is gaining interest in industries such as tooling, which is mainly due to the possibility to manufacture complex shapes, which allow for the fabrication of intricate internal cooling channels, and at the same time to achieve fine microstructures. Another key of metal AM for these applications is the possibility of reducing post-processing costs and lead time during the tool manufacturing.

Powder bed fusion (PBF) technologies, using both laser and electron beam sources (particularly Selective Laser Melting, SLM, and Electron Beam Melting, EBM [1]), have been used in the attempt to process tool and maraging steels [2,3,4,5,6,7,8,9,10,11]. The main challenge in additive manufacturing (AM) of such materials is their susceptibility for cracking due to the build-up of residual stresses in the melting/solidification stage; this issue is especially pronounced in the case of laser-based AM. In particular, the EBM technology has several characteristics that make it well-suited for processing highly alloyed steels for tooling applications, such as the selective and local heating, high building temperatures, high vacuum during processing, and high building rates [12,13,14]. Within this group of materials, most manufacturing efforts using EBM have targeted hot-work tool steels [2,3,4,6]. On the other hand, there is a lack of investigations focusing on high carbon cold-work tool steels, and just a few reports can be found in the literature, where only one example refers to the use of AM in the production of worm-milling cutters [15,16,17].

In a preliminary study by the authors [18], the EBM processing of a highly alloyed cold-work tool steel (Vanadis 4 Extra) was investigated. Different combinations of process parameters were explored for manufacturing cubic specimens, and the process window for obtaining pore-free material that was free of swelling was achieved. Solid specimens were obtained at area energies between 1.5 and 2.0 J/mm^2^, and the resulting microstructure was found to be composed of fine sub-cells with Mo- and V-rich carbides precipitated mostly along the cell boundaries. Hardness in the as-built condition ranged between HRC 54 and 59, and an adjusted heat treatment was performed allowing for HRC 63.

The aim of this investigation is to evaluate key mechanical properties of a high carbon cold-work tool steel manufactured using EBM. In doing so, larger specimens (bars and cylinders) were manufactured in order to produce mechanical test specimens. Key mechanical properties for the targeted tooling applications such as hardness, compressive strength, un-notched impact toughness, and pin-on-disc wear properties were investigated. In addition, the present work also aims for the design of a set of heat treatment protocols to generate an optimized microstructure and to achieve the targeted mechanical properties. Hot isostatic pressing was also applied to selected specimens in order to further improve the as-built impact toughness and to investigate its effects on the final microstructure and mechanical properties.

## 2. Materials and Methods

### 2.1. Powder

A highly alloyed cold work tool steel by Uddeholms AB (Hagfors, Sweden), benchmarked as Vanadis 4 Extra [19], was used as a feedstock material. This steel has a high carbon, chromium, molybdenum, and vanadium content (C:1.4; Cr: 4.7; Mo:3.5 and V: 3.7 in weight %). The material was gas atomized and then sieved to a size fraction of 50–150 µm. Flowability of the powder in the Hall flow meter is 13.4 s/50 g, and its apparent density is 4.42 g/cm^3^. Detailed information regarding powder composition and properties can be found elsewhere [18,19].

### 2.2. EBM Processing

**Figure 1:**
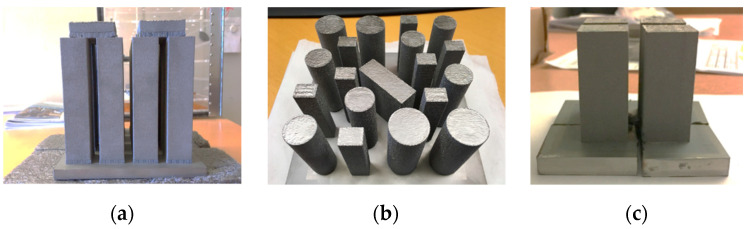
Bars and cylinders for mechanical test. First (**a**), second (**b**), and third (**c**) build jobs.

Three build jobs composed of bars and cylinders (as shown in Figure 1) were prepared in order to machine mechanical test specimens. The first and third build jobs were manufactured in an Arcam A2 system, whereas an Arcam Q1 (both by ARCAM EBM, Mölnlycke, Sweden) was used for the second build. Parts were melted using only hatching in a cross-snake strategy (no contours), with a layer thickness of 50 µm and a process temperature of 850 °C. Specimens were built with area energies between 1.5 and 2.1 J/mm^2^ using a line offset of 0.1 mm.

### 2.3. Heat Treatment

Heat treatment was performed with two purposes: to ease the machining process needed to obtain the mechanical testing specimens and to optimize the as-built material microstructure after the EBM process. Before the machining, a soft annealing at 750 °C for 4 h was applied to bars and cylinders both in the as-built condition and after HIP treatment for selected specimens. For microstructure optimization, different heat treatment protocols were designed and carried out after successive iteration loops, based on the analysis of the microstructure, hardness, and levels of retained austenite. The austenitization of specimens was performed at temperatures between 1020 and 1200 °C, and the quenching was carried out in vacuum. In some of the heat treatment protocols explored, vacuum stage was followed by deep cooling at −196 °C. Two of the heat treatment protocols leading to desired material properties were further applied to the specimens prior to mechanical testing.

### 2.4. Hot Isostatic Pressing

Hot isostatic pressing (HIP) was performed to selected specimens aiming to increase the ductility of the as-built material by eliminating processing defects and to investigate how HIP affects the mechanical properties of the material. The HIP cycle was applied at 1160 °C with a heating rate of 10 °C/min and a holding time of 30 min at 200 MPa. Specimens were quenched with an ultra-rapid quenching (URQ) to 60 °C. The HIP process was carried out in a Quintus QIH 48 (by Quintus Technologies AB, Västerås, Sweden). Then, HIPed specimens were subjected to the selected heat treatment protocols.

### 2.5. Microstructure and Mechanical Characterization

Scanning electron microscopy (FEI Quanta 600F, Philips/FEI, equipped with both secondary and back-scattered electron detectors, Hillsboro, OR, USA) and energy-dispersive X-ray spectroscopy (EDS, Tescan Maia 3, Tescan, Brno, Czech Republic) were used to characterize the microstructure and analyze the elemental composition of the processed material. EDS data were acquired and analyzed using AZtec software (Oxford Instruments, Abingdon, Oxfordshire, UK). Carbide content and retained austenite of the specimens were studied by a Seifert™ XRD 3003 PTS system with a PSD detector and a Cr source at an angle of rotation φ = 360° and an oscillation angle θ = 130°.

Critical mechanical properties such as hardness, compressive strength, unnotched impact toughness, and pin-on-disc were investigated. Similarly, microstructure, chemical composition, and retained austenite levels were characterized. Rockwell C hardness was evaluated in a Rockwell hardness meter (Zwick Roell ZHR, Ulm, Germany) using a diamond cone indenter at a 150 kgf load. The pin-on-disc test was performed at a universal testing machine by following the procedure of ASTM G99-17 [20]. The pins were machined from the EBM manufactured specimens, and Al_2_O_3_ (800 mesh) and SiC (150 mesh) abrasive papers were used against the pins. Tests were performed under dry conditions, and samples were ground down to a surface roughness of Ra: 0.5. Wear results were calculated by following the guidelines of the ASTM G99-17 standard. The weight of the pins was evaluated by a precision scale with 0.0001 g resolution before and after the execution of the tribotests. Compressive strength was evaluated in a MTS Servohydraulic 1000 kN testing machine according to Uddeholm’s internal standards. Unnotched impact toughness tests were carried out in a Zwick/Roell Amsler 450 J (Zwick Roell ZHR, Ulm, Germany) impact testing machine from prismatic machined specimens according to ISO 148-1 [21] standard. All tests were performed at room temperature.

## 3. Results

### 3.1. As-Built Microstructure

**Figure 2:**
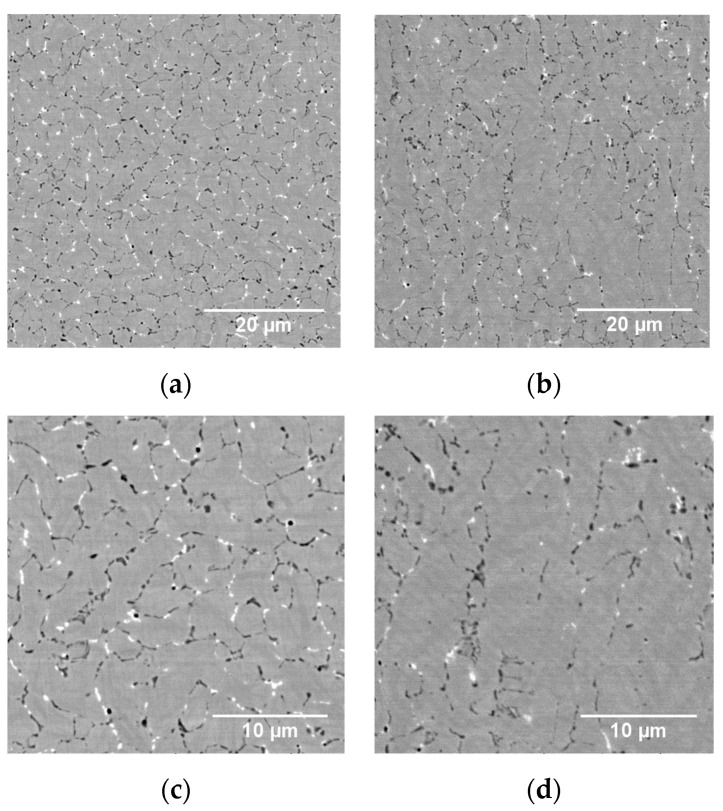
Typical as-built microstructure at low (**a**,**b**) and high (**c**,**d**) magnifications, from cuts perpendicular (**a**,**c**) and parallel (**b**,**d**) to the build direction. Micrographs are acquired in a SEM using BSE contrast.

A typical as-built material microstructure found in cubic specimens is presented in the SEM micrographs of Figure 2. A fine cellular-like structure with carbides precipitated along the cell boundaries can be observed. The microstructure is equiaxed in the build plane (XY-cut in Figure 2a,c), whereas elongated columnar grains growing with the thermal gradient are evident parallel to the build direction (Z-cut in Figure 2b,d).

**Figure 3:**
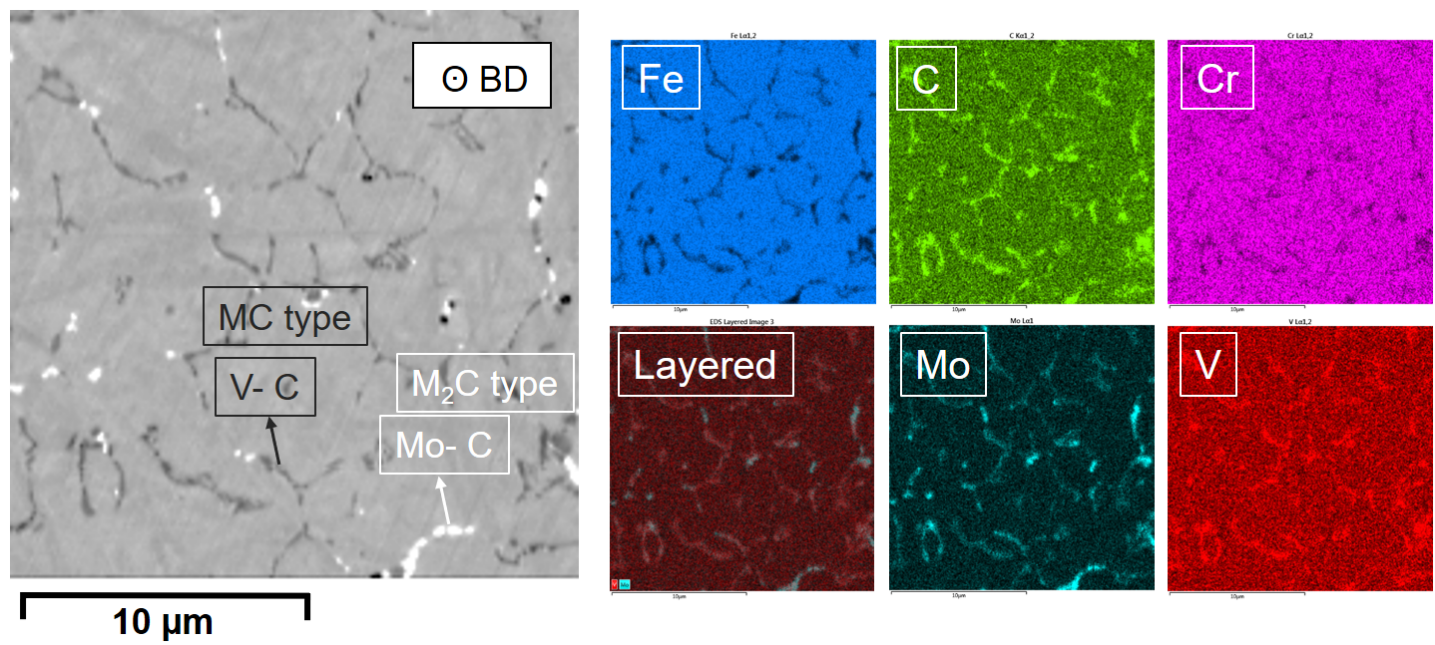
Selected area and compositional EDS color maps showing distribution of different elements present in the alloy.

In order to investigate the nature of the carbides, an EDX analysis was carried out. EDX maps were performed in a 20 µm grid, and the elemental color maps corresponding to the main alloying elements are presented in the images of Figure 3. It is evident that the carbides appearing white in the SEM images under BSE contrast are rich in molybdenum, and most probably are of the M_2_C type, whilst the dark-gray carbides are rich in vanadium, and most probably are of the MC type.

**Figure 4:**
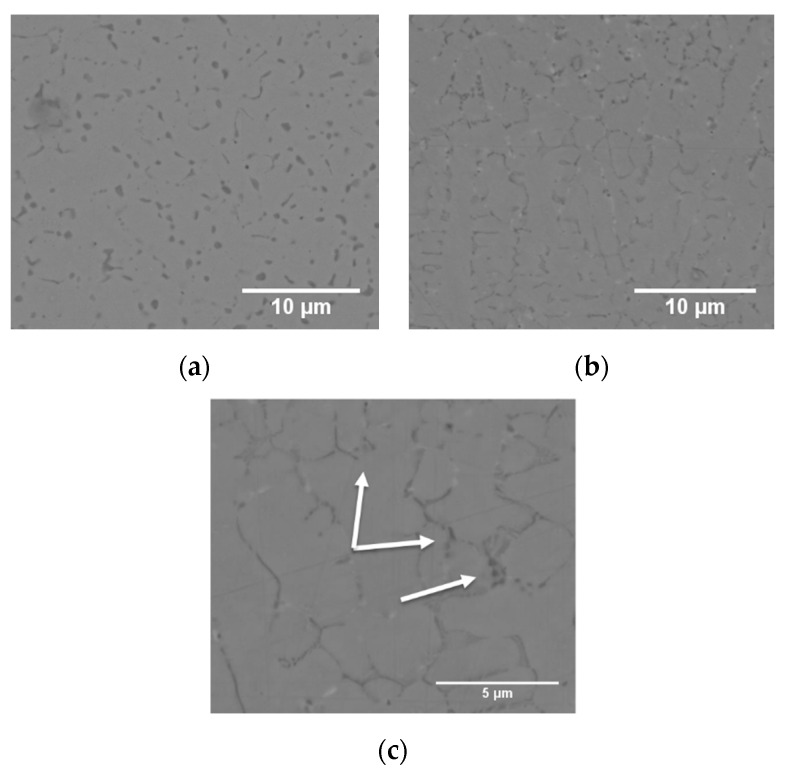
Representative microstructure of build job 1. Cut perpendicular (**a**) and parallel (**b**) to the build direction. Figure (**c**) shows the inter-dendritic eutectic carbides in a sample from a second build job.

In Figure 4a,b, representative micrographs of the microstructures obtained from bars/cylinders for mechanical testing specimens are presented. The same features were found as in the small cubic specimens, with equiaxed grains in the XY-plane and elongated ones in the Z-plane. It is evident that the solidified microstructure and carbide precipitation behavior configure a discontinuous carbide network, while very fine primary carbides are homogeneously dispersed in a martensitic matrix together with over 15% retained austenite (see Table 1). The dendritic solidification nature is revealed by the presence of inter-dendritic eutectic carbides along the cell boundaries, as marked by arrows in Figure 4c.

The as-built hardness and retained austenite levels were investigated for the different build jobs, and the results are presented in Table 1. Consistent hardness values and retained austenite levels were found for the different build job specimens, which are also comparable to the ones found in preliminary investigation for small cubic specimens in the as-built condition [18]. Nevertheless, according to the targeted application of this material in cold-work tools, higher hardness and lower levels of retained austenite are demanded, and therefore, heat treatments need to be developed.

### 3.2. Heat Treatment Development

In order to optimize the as-built material and transform the retained austenite, a set of heat treatments were performed, and the final microstructures were analyzed. Specific heat treatment parameters and conditions, as well as the hardness and levels or retained austenite achieved after the treatments, are presented in Table 2.

**Figure 5:**
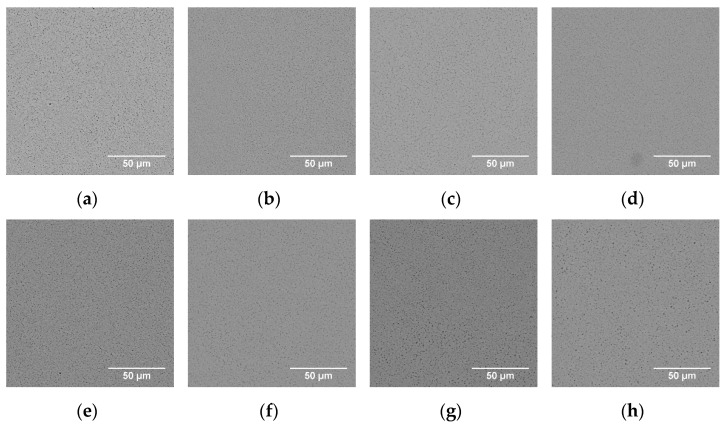
SEM images using BSE detector for the microstructures after the heat treatment protocols HT1 to HT7 (**a**–**g**) and HT7/1 (**h**), correspondingly. Images are acquired for XY-cuts perpendicular to the build direction.

Material microstructures resulting after different heat treatment protocols (1, 7, 7/1) can be observed in the SEM images presented in Figure 5. In general, all heat treatments performed resulted in complete dissolution of unwanted molybdenum carbides found in the materials in the as-built condition. Furthermore, in all cases, the microstructure is transformed to have homogeneous dispersion of vanadium carbides, varying slightly in size and producing a web-type distribution in the matrix.

**Figure 6:**
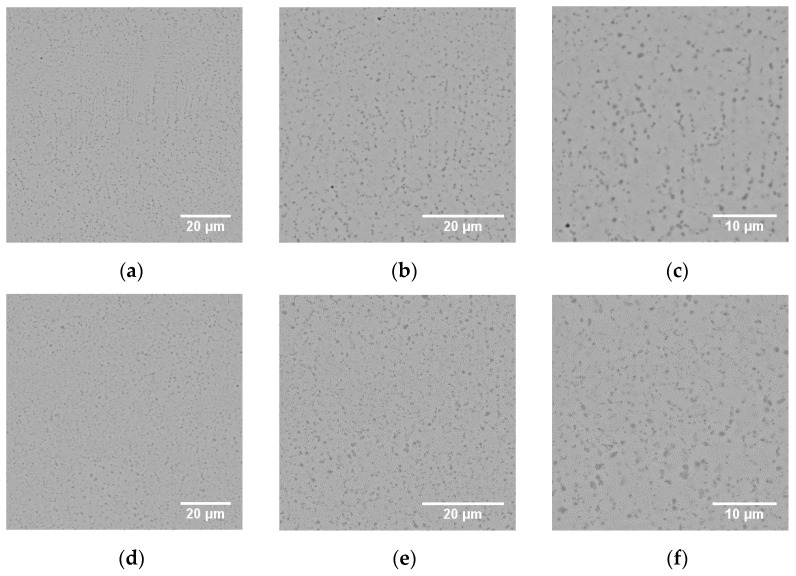
Different magnification SEM images for the heat treatments HT3 (**a**–**c**) and HT5 (**d**–**f**). Images were acquired from cuts parallel to the build direction.

From the hardness values achieved after the heat treatments, and accounting for the microstructural features observed, the protocols No. 3 and 5 (hereafter referred to as HT3 and HT5) were considered as suitable for the application and therefore were further adopted for being applied to the mechanical testing specimens. Different magnification SEM micrographs for HT3 and H5, from cuts parallel to the build direction, are presented in Figure 6.

It can be noticed from the images of Figure 6 that both HT3 and HT5 yield a high-density dispersion of discontinuous and fine V carbides. The columnar character of the cells observed in in the build direction for the as-built condition, where carbides precipitate in the cell boundaries, is yet slightly noticeable after HT3 (Figure 6a–c). On the other hand, HT5 (Figure 6d–f) breaks down the columnar nature of the microstructure, and fine carbides with different sizes and morphologies are homogeneously distributed in the matrix.

### 3.3. Mechanical Properties

Specimens for mechanical testing were subjected to H3 and HT5 prior to the tests. A pin-on-disc test was used to evaluate the abrasive wear, while a compression test was used to determine the yield strength and Young’s modulus. An unnotched impact toughness test was performed to evaluate the ductility.

#### 3.3.1. Abrasive Wear

**Figure 7:**
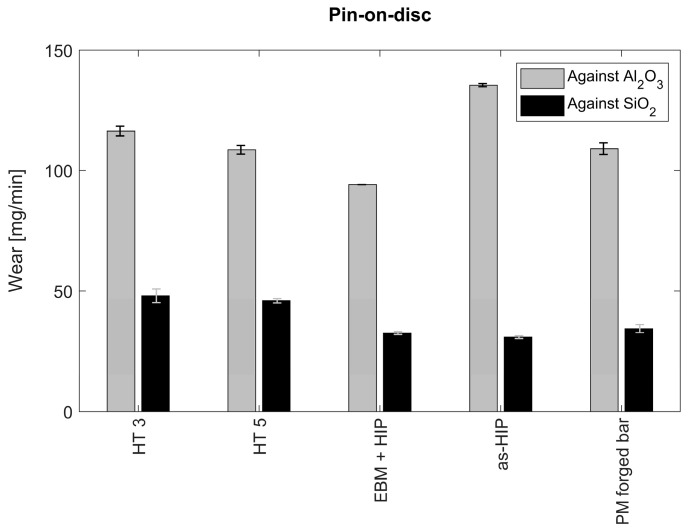
Wear results of pin on disc against Al_2_O_3_ and SiO_2_ abrasive papers. Specimens were manufactured using powder metallurgy before and after forging (As-HIP and PM-forged bars), EBM-manufactured and HIPed (EBM + HIP), and EBM + heat treatment according to protocols 3 and 5 (HT3 and HT5). Hardness corresponds to 62–64 HRC.

Regarding wear, both tests against Al_2_O_3_ and SiO_2_ papers presented abrasive wear rates similar to conventionally produced Powder Metallurgy (PM) Vanadis 4 Extra material, as presented in Figure 7. The obtained values for the EBM-produced, EBM-produced and HIPed, as-HIPed, and conventionally manufactured PM Vanadis 4 Extra material are in the order of 110–115, 94, 135, and 110 mg/min against Al_2_O_3_ paper, and in the order of 48, 45, 32, and 30 against SiO_2_ paper, respectively. It can be noticed that the results for both types of abrasive papers show a similar trend. An HIP effect can be clearly seen; however, EBM-produced Vanadis 4 Extra specimens exhibit a similar performance to PM bar after forging. The HIP material subjected to HT5 showed some better performance, which can be related to the carbide precipitation. Jin et al. found that the M_2_C and MC type carbides are heavily exposed to refinement at the inter-dendritic region, which occurs due to very high cooling rates (10^4^ to 10^7^ °C/s) during the EBM process [22]. It is considered that high solidification rates are characteristic to PBF-AM and EBM in particular, and later on high austenitization is in favor of finer secondary carbide precipitation while better solubility in the matrix occurs. This phenomenon might be the reason for the higher hardness and wear resistance of HT5 as compared to HT3. Additionally, not only the higher austenitizing temperature of HIP but also ultra-high rapid quenching rates might contribute to the constitution of secondary carbide precipitates and finer martensitic microstructure.

#### 3.3.2. Ductility

**Figure 8:**
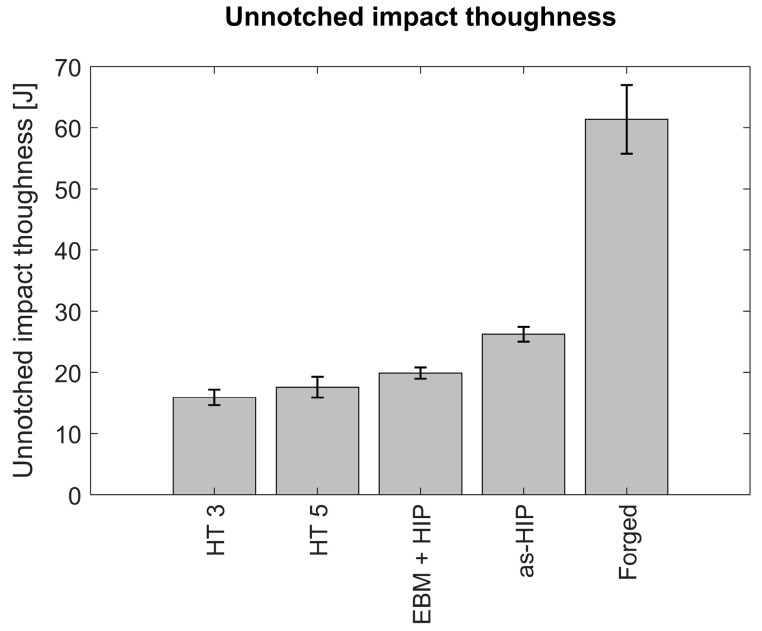
Unnotched impact test results of HT 3 and 5 as compared with conventionally manufactured counterparts. Specimens were manufactured using powder metallurgy before and after forging (As-HIP and PM-forged bars), EBM-manufactured and HIPed (EBM + HIP), and EBM + heat treatment according to protocols 3 and 5 (HT3 and HT5). Hardness corresponds to 62–64 HRC.

Impact toughness up to ≈17 J were reached for specimens after HT3 and HT5. However, for the intended cold-work applications, 20 J are required, and so HIP was used as a way to increase further the ductility. After applying HIP, impact toughness values increased to 21 J. The comparison of the unnotched impact toughness of the specimens with different process routes is presented in Figure 8.

Since the material was not forged bar, an as-HIP property profile was aimed for the EBM-produced material. Therefore, one can say that the target level of ductility was achieved. Nevertheless, the effects of ultra-rapid quenching or a high cooling rate of EBM are unknown and should be further investigated. It is known that the precipitates can facilitate an improvement of the strength, where they might act as cleavage initiation points and decrease the ductility [23,24,25].

#### 3.3.3. Compressive Strength

**Figure 9:**
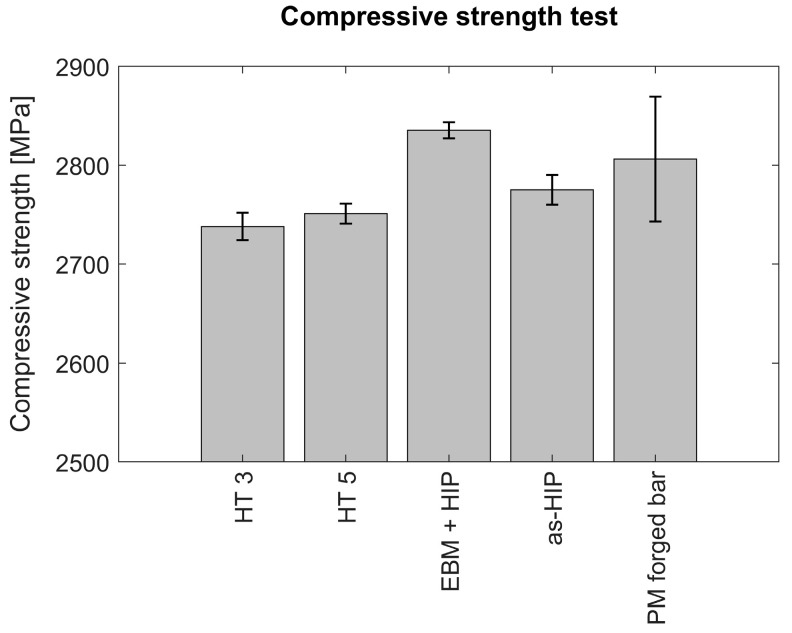
Compressive strength test results of HT 3 and HT5 as compared with conventionally manufactured counterparts. Specimens were manufactured using powder metallurgy before and after forging (As-HIP and PM-forged bars); EBM-manufactured and HIPed (EBM + HIP), and EBM + heat treatment according to protocols 3 and 5 (HT3 and HT5). Hardness corresponds to 62–64 HRC.

The yield strength was found to be around 2724–2761 MPa, and the Young’s modulus was found to be around 225 GPa for EBM-manufactured material, comparable to PM forged bar (Figure 9). Despite the fact that the Young’s modulus is typically not affected, unless a drastic difference in composition occurs, EBM-produced specimens showed a higher Young’s modulus than the forged bar. This was discussed in the work of Speich et al. [24], and the influence of elastic modulus through altering the distribution of electrons was shown as an alternative way to have an influence on the elastic modulus by dissolving the alloying elements substitutionally. Therefore, in this study, the increase in Young’s modulus is speculated to be connected to the distribution of carbides and formation of new phase constitutions in the lattice structure, which will be a scope of further investigations. Additionally, it was seen that HIP is effective on increasing both yield strength and Young’s modulus. It is speculated that the increase in Young’s modulus is due to the distribution of carbides. It is known that the substitutionally dissolved alloying elements have an influence on the elastic modulus [26]. An increase in yield strength together with the low ductility can also be interpreted as a result of finer carbide precipitates and full transformation of retained austenite with the help of rapid quenching [27,28,29].

High hardness together with high yield strength are significant properties for wear reduction in order to extend the tool life. EBM provides higher hardness than conventionally manufactured PM material after austenitization and quenching, which can be a result of fine-grained martensitic and dendritic microstructure that results from the high cooling rates characteristic to EBM and laser-based PBF-AM, as stressed by Junker et al. [30]. The discontinuous carbide network was also considered to be the reason for the relatively high compression strength. Another property that increases the tool life is high ductility. Michaud et al. [31] showed that high mechanical properties can be achieved by having V-rich carbide precipitation. On the other hand, it was also emphasized that discontinuous or heterogeneous distribution of carbides in the matrix can be a limiting factor for fracture toughness and Charpy impact, regardless of the austenitizing and tempering conditions [32]. This can be speculated as another reason to lower ductility values.

#### 3.3.4. Summary of Results

In Table 3, a summary of the results from EBM materials are compared with that of conventionally produced material, i.e., as-HIP (powder metallurgy) and forged (powder metallurgy). Most properties are comparable or slightly higher for the EBM material, and the impact toughness could be increased to 21 J, which is acceptable for the specific application and comparable to as-HIP materials.

## 4. Conclusions

In this work, the microstructure and mechanical properties of Cr-Mo-V cold work tool steel after additive manufacturing by the electron beam melting (EBM) process were evaluated. Based on the results obtained, the following conclusions can be drawn:

1. As-built specimens were manufactured with a microstructure consisting of discontinuous carbides, which is characteristic of eutectic solidification. Inter-dendritic regions showed finer carbide precipitations, predominantly M_2_C (Mo-rich) and MC (V-rich) types.
2. Heat treatments allowed for an optimized microstructure yielding a homogeneous dispersion of discontinuous V-rich carbides and complete dissolution of Mo carbides, reaching up to HRC 65. Retained austenite was measured below 2% for all heat-treated specimens.
3. The compressive strength, hardness, and wear properties of EBM-manufactured samples are superior to as-HIP manufactured products and similar to PM material. However, ductility of the as-built EBM manufactured samples exhibited low values (below 20 J), which was evaluated as a result of internal defects.
4. Hot isostatic pressing (HIP) can be motivated in order to improve the mechanical properties by eliminating internal defects and by the effect of ultra-high rapid quenching with higher cooling rates. Ductility was increased to 21 J, yield strength and Young’s modulus reached up to 2843 MPa and 230 GPa after HIP, respectively.
5. The EBM route provides valuable possibilities for the manufacturing of advanced components from Vanadis 4 Extra for tooling applications.

## Figures and Tables

**Table 1:** Hardness, as measured in the XY direction, and retained austenite in the as-built condition for the different build jobs.

Build Job	Hardness (HRC)	% Retained Austenite
1	55–56	17 ± 4
2	56–58	16 ± 4
3	56–58	17 ± 3

**Table 2:** Different heat treatments and corresponding parameters.

HT	Austenitization		Quenching			Tempering	Hardness	RA
	T (°C)	t (min)	Conditions	t (s)	T (°C)	t (h)	HRC	%
1	1020	30	Vacuum	300	540	4 × 1	60–61	<2
2	1100	30	Vacuum	300	525	4 × 1	60–61	<2
3	1100	30	Vacuum	300	540	4 × 1	63–64	<2
4	1160	10	Vacuum + DC	3600	525	3 × 1	61–62	<2
5	1160	10	Vacuum + DC	300	525	3 × 1	63–65	<2
6	1160	10	Vacuum + DC	10,800	525	3 × 1	61–62	<2
7	1200	10	Vacuum + DC	300	525	3 × 1	62–64	<2
7/1	1020	30	Vacuum	300	525	3 × 1	60–62	<2

**Table 3:** Summary of mechanical properties as compared with conventional HIPed and forged materials.

Property	As-HIP (PM)	Forged HIP, Forged and Heat Treated Conventional PM Route	EBM + HT (Not Forged)	EBM + HIP + HT (Not Forged)
Hardness, HRc	61–63	62–64	62–64	64–65
Retained Austenite, %	<2	<2	<2	<2
Unnotched Impact Toughness, J	≈25	≈60	Up to ≈17	Up to ≈21
Yield strength (MPa) (Compression)	2760–2790 (at 63 HRc)	2743–2869 (at 64 HRc)	2724–2761 (at 64 HRc)	2827–2843 (at 64 HRc)
Young Modulus (GPa) (Compression)	-	≈230 (at 60 HRc)	≈225 (at 63 HRc)	≈230 (at 64 HRc)
Pin on Disc (mg/mm)	≈135	≈110	≈110	≈90

## Data Availability

The data presented in this study are available on request from the corresponding author.
